# Affordable Cardiac Rehabilitation An Outreach Inter-Disciplinary Strategic Study (ACROSS) – Research Programme Protocol

**DOI:** 10.3310/nihropenres.13957.1

**Published:** 2025-05-06

**Authors:** Rod S Taylor, Imran Bashir Chaudhry, Mithila Faraque, Panniyammakal Jeemon, Amy Blakemore, Karina Lovell, Nusrat Husain, Tahir Saghir, Saidur Rahman Mashreky, Sivadasanpillai Harikrishnan, Chaudhury Meshkat Ahmed, Abraham Samuel Babu, Alex McConnachie, Emma McIntosh, Rakhshi Memon, Sally Singh, Alastair Leyland, Bhautesh Jani, Walter Flores, ACROSS Collaborative Group, Rezaul Karim, Zia Ul Haq, Nabila Soomro, Zainab B Zadeh, Ameer Khoso, Sehrish Tofique, Farhat Jafri, Riffat Sultana, Huma Naeem, Palash Chandra Banik, Sohel Choudhury, Louise Taylor, Jamal Uddin, Claire Copping, David Innes, Sunil Roy TN, Stigi Joseph, Vijayan Ganesan, Mukund A Prabhu, Jyothi Vijay MS, Ramya Das NK

**Affiliations:** 1 School of Health and Wellbeing, University of Glasgow College of Medical Veterinary and Life Sciences, Glasgow, Scotland, G12 8TA, UK; 2 Pakistan Institute of Living and Learning, Karachi, Sindh, Pakistan; 3 The University of Manchester, Division of Psychology and Mental Health, Manchester, England, UK; 4 University of Karachi, Karachi, Sindh, Pakistan; 5 Bangladesh University of Health Sciences, Dhaka, Dhaka Division, Bangladesh; 6 Sree Chitra Tirunal Institute for Medical Sciences and Technology, Thiruvananthapuram, Kerala, India; 7 National Institute of Cardiovascular Diseases, National Institute of Cardiovascular Diseases, Karachi, Sindh, Pakistan; 8 Department of Public Health, North South University, Dhaka, Dhaka Division, Bangladesh; 9 Bangabandhu Sheikh Mujib Medical University, Dhaka, Dhaka Division, Bangladesh; 10 Department of Physiotherapy, Manipal College of Health Professions, Manipal, Karnataka, India; 11 University College London, Faculty of Population Health Sciences, London, England, UK; 12 Respiratory Sciences, University of Leicester, Leicester, England, UK; 13 University Hospitals of Leicester NHS Trust, Leicester, England, UK; 14 Accountability Research Center, American University Washington College of Law, Washington, District of Columbia, USA

**Keywords:** cardiac rehabilitation, mental health, coronary artery disease, heart failure, randomised controlled trial, low and middle income country.

## Abstract

**Background:**

The evidence and infrastructure needed to access and deliver cardiac rehabilitation (CR) services are absent or lacking in low and middle-income countries (LMICs), resulting in a substantial loss of potential health and socio-economic benefits. Home-based programmes provide an affordable model of delivery that can leverage a scalable increase in CR access in LMICs. ACROSS (Affordable Cardiac Rehabilitation: An Outreach Inter-disciplinary Strategic Study) seeks to co-develop (with patients, caregivers, clinicians, and service commissioners) a culturally and contextually applicable and affordable home-based programme for people with the multimorbidity of coronary heart disease and/or heart failure with co-existing depression and/or anxiety and evaluate the acceptability, clinical effectiveness, and cost-effectiveness of its implementation in Bangladesh, India, and Pakistan and to determine its scalability and sustainability.

**Methods:**

Four linked work packages (WPs). WP1 (cultural adaptation/refinement of home-based rehabilitation): examine rehabilitation implementation barriers/enablers from multiple stakeholder perspectives and co-develop a feasible and acceptable culturally & contextually adapted home-based programme, extended to take account of co-existing depression and/or anxiety; WP2 (external pilot): assess feasibility/acceptability of the co-developed rehabilitation intervention and study design and processes necessary for a full-scale trial; WP3: (multicentre/multi-country hybrid effectiveness and implementation randomised trial) determine the clinical and cost-effectiveness of a culturally adapted home-based rehabilitation intervention for people with coronary heart disease and/or heart failure and depression and/or anxiety; WP4 (capacity building): build research and rehabilitation delivery capacity.

**Conclusions:**

The ACROSS programme overarching goal is to develop a clinically and cost-effective CR model in low-resource settings for people in Bangladesh, India, and Pakistan with a multimorbidity of heart disease and depression and/or anxiety with the potential for substantial health and socio-economic benefits.

## PLAIN LANGUAGE SUMMARY

The combination of heart disease alongside depression and anxiety is a common and profound unmet need for global health systems, especially in low and middle-income country settings. It affects people of working age, causing them ill-health and major financial loss. Cardiac rehabilitation is recommended as a core component of health services and includes exercise, education, and psychological support. It has been shown to reduce death rate and hospital admissions and improve the quality of life. However, cardiac rehabilitation services in low- and middle-income countries are virtually absent.

Working with partners in Bangladesh, Pakistan, and India, the ‘Affordable Cardiac Rehabilitation: An Outreach Interdisciplinary Strategic Study’ (ACROSS) programme seeks to develop and evaluate the implementation of a clinically effective, affordable, and culturally and contextually appropriate model of home-based rehabilitation. ACROSS has four work packages: (1) Engagement events with those with lived-disease experience, their families, healthcare staff, and service providers to co-develop a culturally and contextually adapted home-based rehabilitation ('ACROSS') programme and training materials for providers; (2) A pilot study to understand if the ACROSS programme is acceptable and practical for patients and staff and whether a full-scale trial is doable; (3) A trial in 3000 patients with multiple long-term conditions that include heart disease and depression and anxiety across several sites in each country to tell us whether the programme improves health outcomes at 12-months and what its affordability is; and (4) Develop a sustainable model of workforce training and implementation to enable long-term programme delivery as well as build partner research capacity.

People with lived-disease experience, clinicians, community leaders, and healthcare policymakers have been extensively involved in the planning of ACROSS. Our stakeholder meetings have consistently emphasised the importance of this research and supported the development of our research plans. Patients and stakeholders in each partner country will provide ongoing advice.

## Introduction

### Background

In January 2024, the National Institute for Health and Care Research (NIHR) on Interventions for Global Health Transformation (RIGHT) programme approved the funding of ‘Affordable Cardiac Rehabilitation: An Outreach Inter-disciplinary Strategic Study (ACROSS)’. ACROSS is a global research programme that seeks to address the question of whether the implementation of an adapted home-based rehabilitation programme for people with multimorbidity that includes coronary heart disease and/or heart failure with a co-existence of depression and/or anxiety in Bangladesh, India and Pakistan, is clinically effective and cost-effective compared to usual care alone. This paper presents the rationale, study design and protocol for the ACROSS programme.

### Rationale

***Burden of heart disease & a mental health disorder.*** Cardiovascular disease (CVD) is the most prevalent non-communicable disease, the leading cause of global mortality, and a major contributor to premature disability and ill-health. CVD causes an estimated 17.8 million deaths worldwide, corresponding to 330 million years of life lost and another 35.6 million years lived with disability ^1, 2^. By 2030, it is estimated that >80% of CVD-related disability and death will occur in low and middle-income countries (LMICs) due to increasing risk factors (e.g., hypertension, smoking, diabetes, obesity) ^2, 3^. South Asia contributes to the highest proportion of CVD burden compared to other regions globally ^4^. Socio-economic effects are particularly marked in LMICs, where CVD more frequently affects those of working age and is estimated to result in a decrease in gross domestic product of approximately 7% ^5^.

The burden of CVD is further exacerbated by the co-presence of one or more chronic diseases - so-called ‘multimorbidity’ ^6^. A common and burdensome multimorbidity is the combination of heart disease and depression or anxiety. Depression and anxiety act both as a risk factor for the development of heart disease and substantially increase the burden of heart disease ^7^. Recent data show depression and anxiety to be highly prevalent in people with heart disease and may even be higher in South Asian populations (e.g., 70.4% depression in heart failure) ^8– 10^. The addition of a mental health problem is associated with a 40–50% increased risk of mortality and major cardiac events and a 30–50% reduction in health-related quality of life (HRQoL) in people with heart disease ^11, 12^.

***Need for cardiac rehabilitation (CR).*** Cardiac rehabilitation (CR) is a complex secondary prevention intervention that aims to improve HRQoL, optimise functioning and disease self-management, and minimise the risk of recurrent cardiac events in people with heart disease ^13^. It comprises of core components that include exercise training, medication management, risk factor management, and psychological support delivered by multidisciplinary teams, including nurses, physiotherapists, dieticians, and physicians.

There is strong evidence supporting the effectiveness and safety of CR programmes, especially for people with CHD and heart failure. The 2021 Cochrane review of 85 randomised trials of CR in CHD (23,430 patients) reported a 12% reduction (relative risk (RR): 0.88; 95% confidence interval (CI): 0.68 to 1.04) in cardiovascular mortality and a 42% reduction (relative risk: 0.58; 95% CI 0.43 to 0.77) in hospital admissions compared to usual care ^14^. The 2024 Cochrane review of 60 randomised trials of CR in heart failure (8,728 patients) shows a 31% reduction (RR 0.69; 95% CI: 0.56 to 0.86) in hospital admissions ^15^. Both Cochrane reviews show CR participation is associated with substantial improvements in exercise/functional capacity, patient HRQoL, and mental well-being ^14, 15^. For those who develop a mental health diagnosis before or following their cardiac event, meta-analyses have shown significant and consistent improvements in their levels of depression and anxiety following CR participation ^16^.

Despite its clinical and cost-effectiveness and international guidelines recommending its use, CR remains globally underutilised ^17^. This is particularly the case in LMICs, where there are typically few CR programmes due to a lack of public health funding and healthcare professional capacity ^18, 19^. An international survey published in 2019, reported that some 50% of countries currently have CR programmes, with the lowest availability in LMICs ^19^. Similar findings were also observed in the Southeast Asian region ^20^. Furthermore, most evidence supporting CR has come from high-income countries ^14, 15^.

***The potential of home-based CR delivery.*** Given constraints in their healthcare infrastructure, LMICs require affordable, scalable, and context-appropriate CR approaches ^21^. These include home and community-based programmes supported by accessible digital technologies (e.g., internet and mobile phones) and training for healthcare staff to ensure quality of CR delivery. Home-based programmes can also reduce barriers to CR access and uptake, reducing the need for travel to hospital centres and flexibility to allow rehabilitation to fit around employment commitments ^17^.

There is strong evidence that home-based programmes delivering the core components of rehabilitation and supported by healthcare staff can improve patient outcomes to those seen in more traditional centre/hospital-based programmes ^22^. For example, the REACH-HF (Rehabilitation EnAblement in Chronic Heart Failure) trial in the UK showed that home-based rehabilitation significantly improved HRQoL and was cost-effective ^23, 24^. The adjunct use of mobile technology (e.g., app-based delivery of education material/wearables such as accelerometers to track behaviour changes/web-based consultations) to a home-based programme provides a key opportunity to further enhance rehabilitation access and cost-effectiveness ^25^. The COVID-19 pandemic resulted in a sea change in CR delivery ^26^. Since then, CR has pivoted to home-based models and mobile technology (phone and web consultations replacing home visits plus provision of online facilitator training for healthcare staff) ^26, 27^.

An interdisciplinary programme of implementation research is now needed to test whether a culturally and contextually appropriate affordable home-based delivery model of rehabilitation can be developed (alongside the necessary increases in healthcare staff delivery capacity) to be sufficiently scalable and sustainable to meet the vast current unmet secondary preventive burden of LMICs. A cost-effective CR model in low resource settings for people with multimorbidity that includes the high unmet need of heart disease, and a mental health condition has the potential to accrue substantial health and socio-economic benefits. The wider implementation of such an alternative affordable rehabilitation model also offers an important strategic opportunity to overcome suboptimal rehabilitation access and uptake experienced in high-income settings.

## Protocol

### Aims and objectives

The overarching aim of the ACROSS programme is the evaluation and implementation of a sustainable and scalable culturally and contextually adapted and affordable model of home-based rehabilitation for people with the multimorbidity that includes heart disease and a mental health disorder.

Specific research objectives:

1. To co-design with key stakeholders a culturally and contextually adapted affordable home-based rehabilitation (‘ACROSS’) programme with low intensity inventions for depression and anxiety (Work Package 1 (WP1)).
2. To co-design ACROSS delivery training for health professionals including a train-the-trainer programme to enable the cascade of training (WP1).
3. To assess the feasibility and acceptability of implementing the ACROSS programme and trial design and examine the parameters and processes of a full-scale trial, including recruitment, randomisation, and retention (WP2).
4. To determine the clinical effectiveness (hierarchical primary outcome of mortality, hospital admission, and health-related quality of life) of the ACROSS programme (WP3).
5. To determine cost-effectiveness of the ACROSS programme in terms of an incremental cost per quality adjusted life year (WP3).

Cross-cutting objectives:

6. To build research and clinical delivery capacity and capability and support south-south partnership and learning to underpin the scalable and sustainable country-wide provision of rehabilitation services (WP4).
7. To embed community and stakeholder engagement to inform the route to impact of the project outputs and findings (WPs1–4).

**Figure 1.**
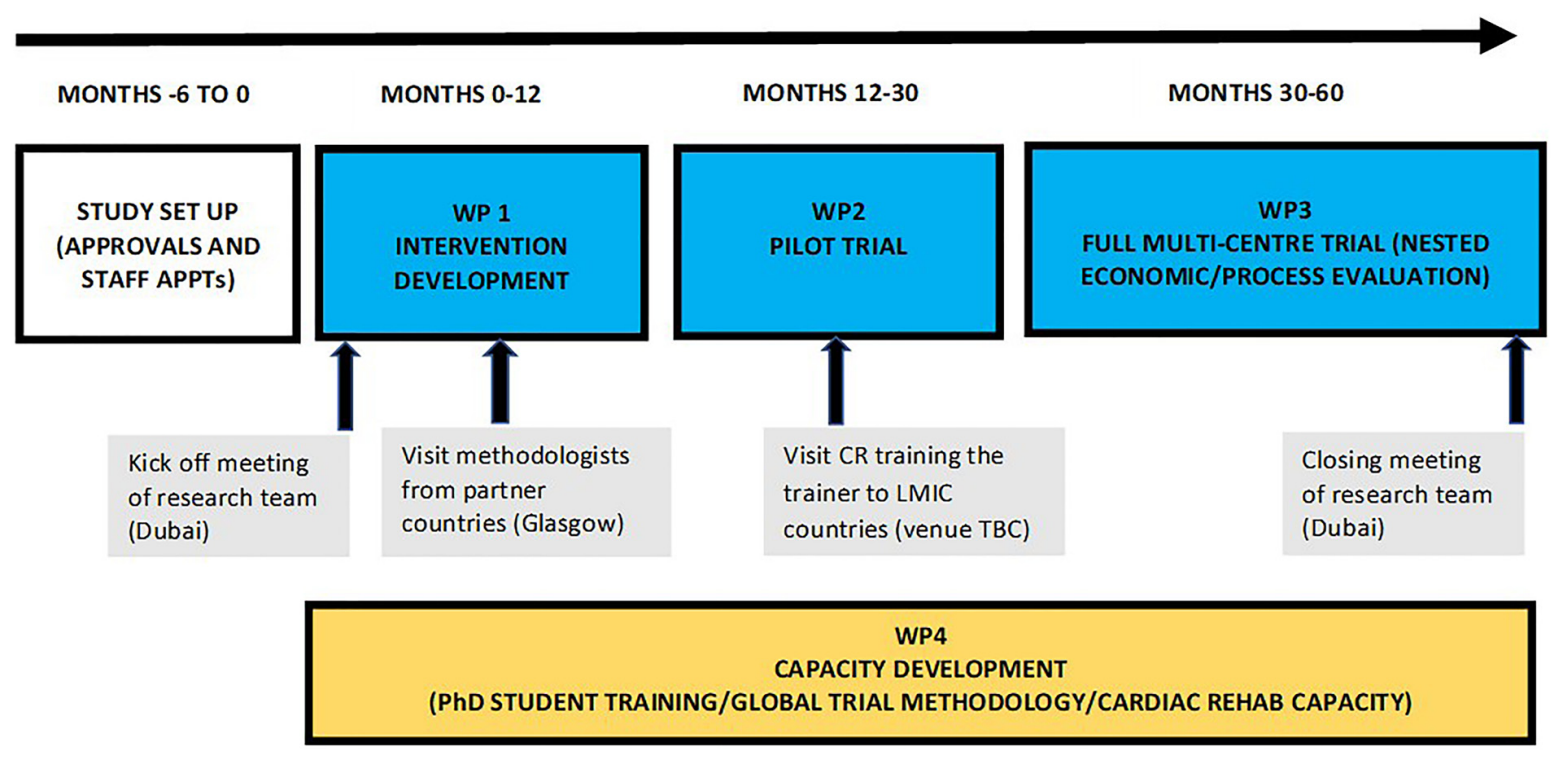
Summary of ACROSS WP.

Through our PhDs (see details in WP4 ‘Training & Capacity Building’ section below), to embed specific methodological research questions into the ACROSS programme. e.g., adapting complex intervention/co-design & co-development of interventions in the LMIC settings. Figure 1 provides a graphical summary of the timing and sequencing of the ACROSS WPs.

## Methods

All research will be performed in accordance with the principles stated in the Declaration of Helsinki. Prior to starting the programme of research (Q2/3 2025), ethical approval will be obtained for all individual protocols from the local institutional review board (IRB) or other appropriate ethics committee to confirm the study meets national and international guidelines for research on humans. Written participant informed consent for all studies will be obtained.

### Patient and Public Involvement

From the outset of the ACROSS programme, we have embedded Community Engagement and Involvement (CEI)/Patient and Public Involvement and Engagement (PPIE) groups in each of our three LMIC countries. Our research plans (research questions/design and conduct of the study/outcomes measures/recuitment strategy) have been developed alongside patients and the public in our partner countries. Our communications manager will coordinate a project dissemination plan with our partner PPIE/CEI stakeholder and patient groups. All materials will be available in local languages and given potential challenges of patient/family literacy, we will seek to ensure materials are appropriately visual and to improve accessibility.

### WP1 - Cultural and contextual adaptation of rehabilitation intervention

Adapting and implementing existing evidence-based interventions to new contexts can introduce new services into new environments that are cost-effectively and timely, with reasonable prospects of successful outcomes ^28^. A goal of adaptation is to maintain the effectiveness of the intervention by preserving core features that account for success while delivering an intervention that is responsive to the new cultural and contextual setting.

WP1 aims to co-design and co-adapt a feasible, culturally acceptable and contextually adapted, affordable home-based rehabilitation (‘ACROSS’) programme (based on Heart Manual ^29, 30^ and REACH-HF ^23, 24^) developed and proven in the UK and tested in principle in a single centre trial in Bangladesh ^31^. It will now be adapted for people with CHD or heart failure in Bangladesh, India, and Pakistan and extended to take account of multimorbidity, including depression and anxiety. We will incorporate and co-adapt low-intensity cognitive behaviour therapy interventions for depression and anxiety (including behavioural activation/cognitive restructuring/problem solving) into the Heart Manual and REACH-HF interventions. In addition, we will develop a facilitator training programme for healthcare practitioners and an associated train-the-trainer programme, to enable the upscaling of delivery capacity.

Objectives:

- Examine the population, patient, and provider contexts in which the home-based rehabilitation would be delivered in our three partner countries.
- Co-design and co-adapt a feasible, culturally acceptable and contextually adapted home-based rehabilitation (‘ACROSS’) programme developed for people with multimorbidity that includes coronary heart disease and heart failure and depression or anxiety.
- Co-design an interactive facilitator training package that can be delivered remotely to ensure scalability and sustainability of the ACROSS programme delivery.
- Co-produce a train-the-trainer facilitator and supervision package for ACROSS.

To achieve our objectives, we will conduct two interrelated studies:

1. Study 1A - Patient and Provider Contexts: This qualitative study will explore implementation barriers and enablers through semi-structured interviews with national health informants and focus groups with health professionals and patients. The aim is to understand the local healthcare contexts and prepare for a broader trial. The Consolidated Framework for Implementation Research (CFIR) ^32^ will guide data collection, focusing on factors like the perceived advantages of the intervention, leadership engagement, and existing care pathways.
2. Study 1B - Intervention Co-Design: This study will use Normalisation Process Theory ^33^ to co-design the ACROSS rehabilitation programme. Collaborating with patients, healthcare workers, and professionals, the team will adapt existing interventions and create new training resources. Consensus processes will ensure the programme aligns with the cultural and contextual needs of the target population.

### WP2 - External pilot trial

WP2 aims to determine the feasibility/acceptability of the ACROSS intervention (co-designed in WP1) and study design and to gather information to examine the parameters and processes of a full-scale trial (WP3), including recruitment, randomisation, and retention.

Objectives:

- Assess participant recruitment and retention.
- Assess the ACROSS programme feasibility and acceptability, exploring barriers and facilitators to uptake and engagement from both participant and healthcare provider perspectives.
- Assess the fidelity and reach of the ACROSS programme and, if necessary, further refine the intervention.
- Assess the feasibility and acceptability of data collection tools and obtain estimates of key cost drivers.
- Assess whether progression criteria are met, and a full randomised trial (WP3) is warranted.

Study Design: A randomised external pilot study with embedded process and economic evaluations. The overarching aim of the external pilot is to implement the co-adapted ACROSS programme developed in WP1 across two sites in each of Bangladesh, India, and Pakistan and to assess the feasibility of a full randomised trial (WP3).

Target population & Setting: see WP3.

Sample Size and Randomisation: To address the objectives of this pilot trial, 100 patients ^34^ from two centres in each of the three partner countries will be recruited over 5 months and randomised as outlined in WP3.

Intervention: ACROSS programme (see WP1), alongside usual care.

Control: No rehabilitation; usual care alone.

*Outcomes & Progression Criteria:* Key outcomes will focus on whether progression criteria are met to proceed to a definitive trial. Patient data will be collected at baseline (pre-randomisation) and 4 months post-randomisation, and economic evaluations will measure key cost drivers like healthcare utilisation and social care.

Process Evaluation: This will assess the feasibility and acceptability of the intervention and study design, following MRC guidelines ^35^. Data will include fidelity checks of intervention delivery, interviews with patients and staff, and analysis of barriers to engagement ^36^.

Data Analyses: The study will be reported according to the CONSORT extension for pilot and feasibility studies ^33^. Quantitative data will be analysed descriptively, while qualitative data will undergo thematic analysis. Themes related to intervention delivery will be interpreted through Normalisation Process Theory (NPT) ^37^.

### WP3 - Hybrid effectiveness-implementation trials of ACROSS rehabilitation

This multicentre, multi-country study evaluates the clinical and cost-effectiveness of the ACROSS rehabilitation programme for individuals with multimorbidity, including heart disease and mental health disorders, across three South Asian countries. It also investigates factors influencing wider implementation in low- and middle-income countries (LMICs).

Objectives:

1. Assess the clinical effectiveness of ACROSS alongside usual care compared to standard treatment alone.
2. Evaluate cost-effectiveness in the three participating countries.
3. Conduct an implementation analysis to understand scalability.

Study Design: Type 1 hybrid effectiveness-implementation randomised controlled trial (RCT) with an embedded economic evaluation ^37, 38^. Participants will be individually randomised (1:1) to receive ACROSS plus usual care or usual care alone. The study will take place in publicly and privately funded healthcare settings.

Participants & inclusion criteria: Adults (≥18 years) hospitalised for acute myocardial infarction, unstable angina, coronary intervention, or heart failure (ejection fraction <45%) with co-existing mild-to-severe depression (Patient Health Questionnaire-9 [PHQ-9] score ≥5) and/or anxiety (General Anxiety Disorder-7 [GAD-7] ≥5) are eligible ^39, 40^. Exclusions include contraindications to exercise, advanced dementia, or conditions preventing safe home-based rehabilitation.

Intervention & Control: It is anticipated that intervention CHD patients will receive an adapted ACROSS version of the Heart Manual intervention ^29– 31^, and heart failure patients will receive an adapted ACROSS version of the REACH-HF intervention ^23, 24^. The final details of the ACROSS program intervention will be confirmed following an extensive co-design process (in WP1) and feasibility testing (in WP2). All participants, including controls, will receive usual medical management by national guidelines.

Primary outcome: A composite of mortality, hospital days, and HRQoL (HeartQoL questionnaire) at 12 months, and analysed using the Win Ratio method ^41, 42^. To calculate the Win Ratio, each intervention group participant is compared with each control group participant. If the intervention group participant has a “better” outcome, it is called a “win”, whereas if the control group participant does better, it is a “loss.” Otherwise, it is a “tie”.

Secondary & implementation outcomes: Includes individual elements of the primary outcome, depression, anxiety, exercise capacity, physical activity, risk factors, and safety events. Implementation outcomes assess reach, adherence, and acceptability.

**Figure 2.**
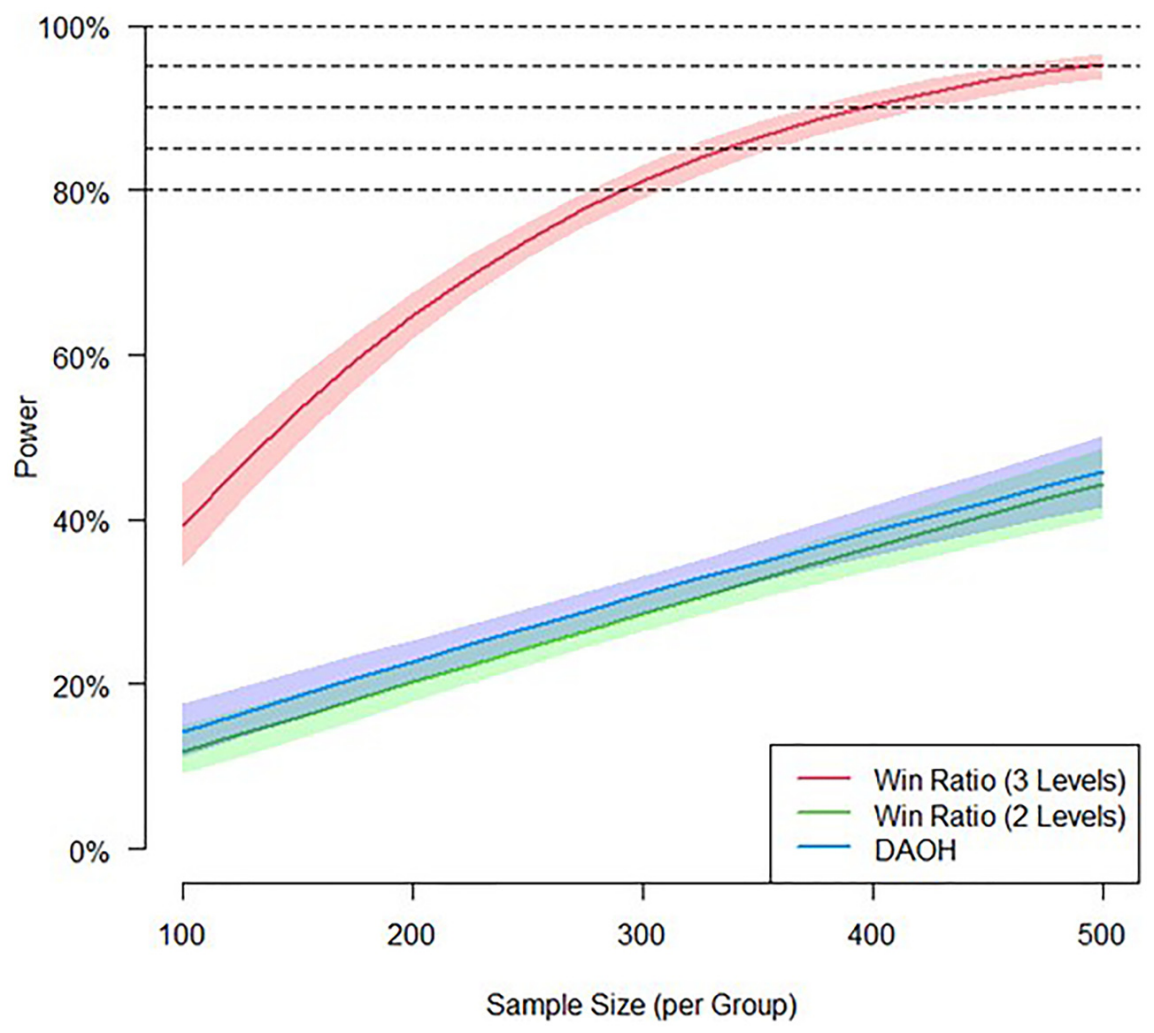
Sample size estimation for WP3.

Sample size: the study employs a simulation-based approach for sample size calculations in Win Ratio analyses, assuming a 12-month mortality rate of 13% and hospitalisation rate of 0.2 per person per year. The intervention is expected to reduce all-cause mortality (hazard ratio 0.85) and hospitalisations (incidence rate ratio 0.75) ^14, 15^. Simulations indicate that a two-level Win Ratio analysis provides comparable power to a non-parametric analysis of days alive out of hospital (DAOH). However, achieving sufficient power requires incorporating health-related quality of life improvements as third level (see Figure 2). A sample size of 1000 patients per country ensures >89% power, accounting for a 20% loss to follow-up.

Recruitment: will span 12 months, with a 4-month internal pilot to check recruitment feasibility, aiming for 336 participants per country. Data collection will take place at baseline, and 4- and 12 months post-randomisation.

Data analysis: The Statistical Working Group will develop a common Statistical Analysis Plan, which will be approved before unblinded analysis. Primary analyses will estimate the Win Ratio considering mortality, hospital days, and HRQoL. Additional regression models and subgroup analyses will be conducted. Missing data will be handled through multiple imputations in sensitivity analyses.

Economic evaluation: will assess cost-effectiveness in low-resource settings, incorporating direct and indirect costs using EQ-5D-5L data. Sensitivity analyses will explore cost variations. Within-trial results will be reported and presented as an incremental cost-utility ratio with the joint distribution of cost/utility pairs being represented on the cost-effectiveness plane and with a cost-effectiveness acceptability curve ^43^ employing cost-effectiveness thresholds based on country per capita gross domestic product (GDP) values and benchmark values (e.g., World Health Organisation [WHO]). Analyses will explore cost effectiveness in subgroups as defined earlier. Recent guidance on conducting economic evaluations in LMIC settings reported by the National Institute of Care Excellence (NICE) International ^44^ will be adhered to as well as reporting standards such as the Consolidated Health Economic Evaluation Reporting Standards statement ^45^.

Qualitative analyses: will assess intervention fidelity and contextual factors through interviews and thematic coding.

### WP4 - Capacity building

The primary goal of WP4 is to enhance research capacity in our LMICs and foster knowledge exchange between the UK and LMICs. The ACROSS project partners with three organisations: the International Council of Cardiovascular Prevention and Rehabilitation (ICCPR), the Heart Manual Office UK, and the Global Health Network (TGHN). These organisations provide expertise in online rehabilitation training and global health research support.

Objectives:

- Supporting PhD studentships.
- Building research and rehabilitation delivery capacity in partner countries.
- Enhancing understanding of complex intervention trials in LMICs.
- Developing a virtual training hub hosted on the ACROSS website.

Key challenges identified by LMIC partners involve the need for training in large-scale research, particularly RCTs, and scaling up rehabilitation services. The project aims to develop equitable partnerships, enabling knowledge transfer between the UK and LMICs and facilitating south-to-south learning. The project will follow the WHO’s ESSENCE principles to strengthen research capacity ^46^.

WP4's training plan includes both face-to-face and virtual activities. In-person training activities will involve researchers from LMIC countries visiting the UK for methodological training and UK trainers visiting LMIC countries to run rehabilitation training and ‘train the trainer’ courses. To maximise accessibility and minimise the carbon footprint of ACROSS, these activities may be adapted to virtual formats. A core element of WP4 is to establish a virtual training hub, providing access to online materials and courses in global health research.

The capacity building efforts already underway include several virtual events including workshops on research theory, mental health forums, and seminars on adapting interventions. ACROSS will promote accreditation for training to enhance the professional development of participants and facilitate future research collaborations.

Research and methodological training will focus on building capacity for RCTs and intervention development, which is essential for the project’s other work packages. This will include training approximately 45 researchers from partner countries in areas including patient-public involvement (PPI), qualitative research, trial methods, health economics, and data analysis. The Glasgow Clinical Trial Unit will coordinate the training and will be supported by materials from global health research networks, including the MRC Research Trials Methodology Partnership.

Rehabilitation service capacity will be built through partnerships with ICCPR and the Heart Manual Office, which will deliver training on home-based rehabilitation programmes. This will include an online ‘train the trainer’ programme, enabling sustainable rehabilitation practices.

A key part of WP4 is the PhD training programme, supporting 8 studentships across the three LMIC countries. These students will work on projects related to the ACROSS programme, global trial methodology, mental health, and rehabilitation. The PhD students will be supported by local LMIC and UK supervisor teams, with access to training materials from the ACROSS hub and opportunities to attend webinars and workshops. To support postdoctoral research, WP4 plans to include activities such as grant writing workshops to help early career researchers submit high-quality grant applications.

### Community engagement & public involvement

Community engagement involvement/patient public involvement engagement (CEI/PPIE) has been embedded into the ACROSS research programme development. Going forward we will seek the following key areas of CEI/PPIE direction: (i) participation in co-development/co-adaptation of our home-based rehabilitation intervention/training materials (WP1); (ii) ongoing advice on all aspects of our research including development of all participant facing documentation (e.g. participant information leaflets), participant recruitment strategies, patient interview guide, and interpretation of qualitative data, writing lay summaries etc; (iii) direction on dissemination/targeting of impact, and (iv) increasing community awareness about comorbidity and decreasing stigma attached to mental health conditions.

Our partner countries have each established local CEI/PPIE that includes: (i) ‘stakeholder group’: regional/national healthcare policymakers, key clinical opinion leaders, representatives of key healthcare professional groups (nursing/physiotherapy/ cardiology/psychology/psychiatry), community/regional leaders (including religious scholars), and senior healthcare providers/managers and (ii) ‘patient involvement group’: people with lived experience of heart disease and depression/anxiety and their families, facilitated by one of our local project team with fluency in local language(s). We expect our CEI/PPIE groups will meet on 3-4 occasions/year, with meetings facilitated by an experienced CEI/PPIE researcher from each country team.

To identify any gaps in skills/training CEI/PPIE contributors may require, we will develop/deliver an induction programme (as part of WP4) to clarify roles/expectations and build trust and rapport with the group members.

**Figure 3.**
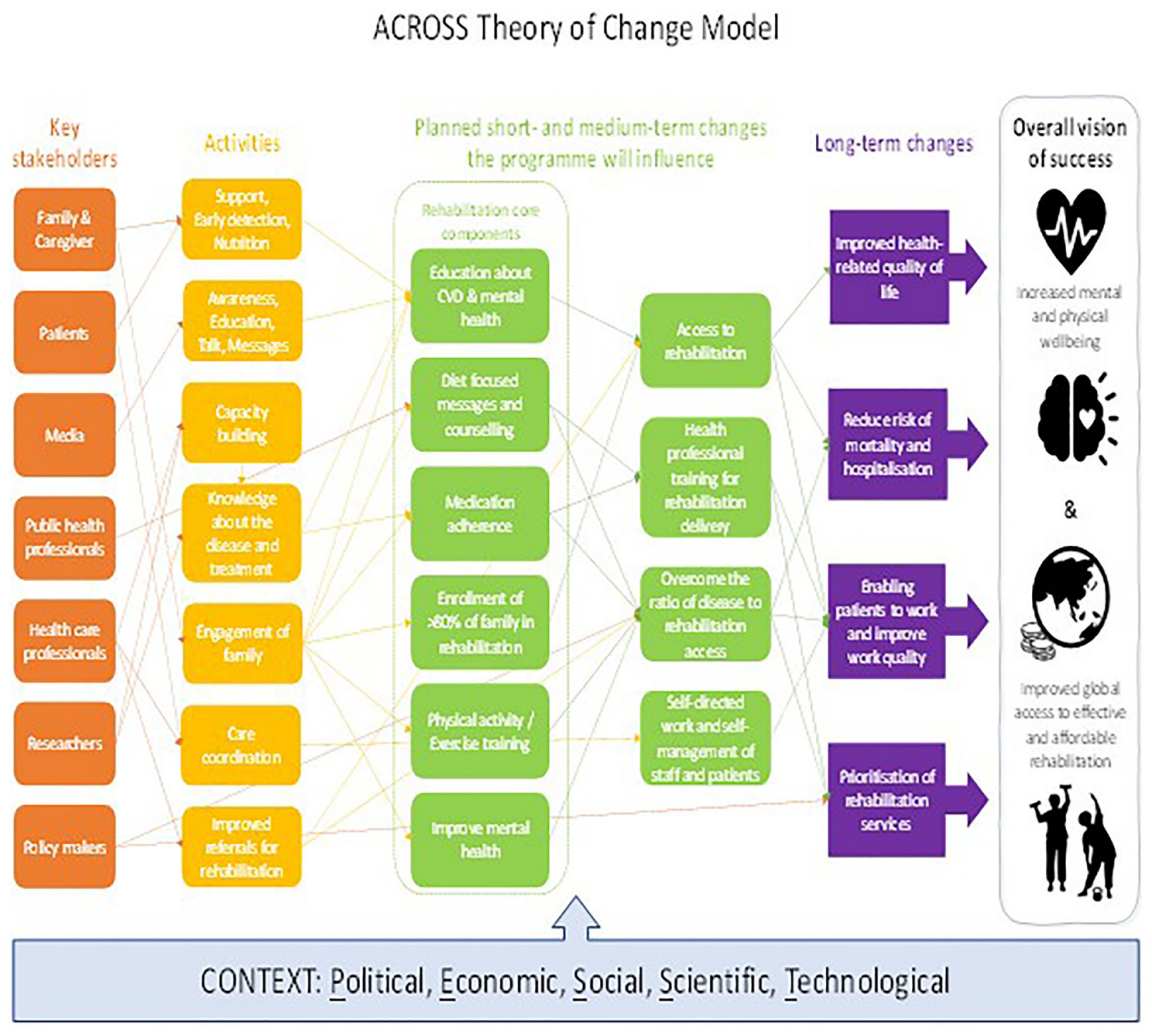
ACROSS Theory of Change model.

Together with our CEI/PPIE group we have developed the ACROSS programme theory of change (see Figure 3).

## Conclusions

The overarching aim of the ACROSS programme to develop a clinically and cost-effective CR model in low resource settings for people with a multimorbidity of heart disease and depression and/or anxiety, offers huge potential health and socio-economic benefits in Bangladesh, India, and Pakistan. The wider implementation of such an affordable intervention model also offers an important strategic opportunity to overcome the problem of suboptimal rehabilitation access experienced in other low- and middle-income countries. We will seek to disseminate our findings widely, using a variety of approaches, supported by our stakeholder and patient advisory groups.

## Data Availability

No data associated with this article.
