# Supplementary material for: Affordable Cardiac Rehabilitation An Outreach Inter-Disciplinary Strategic Study (ACROSS) – Research Programme Protocol
Source: NIHR Open Res. 2025 May 6;5:41. [Version 1] doi: 10.3310/nihropenres.13957.1 (PMC12171719; doi:10.3310/nihropenres.13957.1)
Supplement: Peer review history

# Reviewer report: Reviewer response for version 1

**DOI:** 10.3310/nihropenres.15169.r35712

**Published:** 2025-06-16

**Authors:** Niamh A Merriman

**Affiliations:** 1 School of Public Health, Physiotherapy and Sports Science, University College Dublin, Dublin, Leinster, Ireland

The authors present a well written protocol for a programme of work assessing home-based cardiac rehabilitation in LMICs.

 I would recommend some minor amendments to improve clarity:

**Methods**

 How much of a role will digital technology play in the proposed intervention? Who will be responsible for developing, implementing, and maintaining the digital technologies utilised? Will access to internet in the home be an inclusion criterion? It would be useful to discuss this as both a strength and a limitation.

 The CFIR was updated in 2022, so I suggest using the updated version Damschroder LJ et. al (2022)- ** Ref 1.  ** 

 For WP2 and WP3, usual care can differ massively from one site to another. Do the authors intend to collect detailed information on healthcare utilisation across the control and intervention groups to account for any differences?

 More information on the sample size estimation for the pilot trial is needed.

 What is the timeframe for recruitment to the pilot trial (i.e., how long post-diagnosis) and where will they be recruited from?

 Who will be responsible for recruiting participants to the pilot trial?

 Will the acceptability of randomisation also be assessed?

 Who will conduct the randomisation and how will it be done?

 What are the progression criteria for the full RCT? What if these criteria aren’t achieved?

 The CONSORT statement was updated in 2025, I suggest using the updated version https://www.equator-network.org/reporting-guidelines/consort/

 Will control participants be offered the opportunity to avail of the intervention once the trial is completed?

 Is a further process evaluation planned for the full RCT in WP3? If so, will it follow the same framework as the process evaluation in WP2? More detail is needed here.

**General comments**

 I wish the authors the best of luck with this very worthwhile project.

Is the study design appropriate for the research question?

Yes

Is the rationale for, and objectives of, the study clearly described?

Yes

Are sufficient details of the methods provided to allow replication by others?

Partly

Are the datasets clearly presented in a useable and accessible format?

Not applicable

Reviewer Expertise:

I have expertise in the area of complex intervention development

I confirm that I have read this submission and believe that I have an appropriate level of expertise to confirm that it is of an acceptable scientific standard, however I have significant reservations, as outlined above.

## References

[1] : The updated Consolidated Framework for Implementation Research based on user feedback. *Implementation Science* .2022;17(1) : 10.1186/s13012-022-01245-0 10.1186/s13012-022-01245-0 36309746 PMC9617234

# Reviewer report: Reviewer response for version 1

**DOI:** 10.3310/nihropenres.15169.r35708

**Published:** 2025-06-09

**Authors:** Porkodi Arjunan

**Affiliations:** 1 King Faisal University, Al Hofuf, Saudi Arabia

**Your Report**

 Please provide a full report, expanding on your answers to the questions above. In particular, if you answered “no” or “partly” to any of the questions, please give constructive and specific details as to how the authors can address any criticisms. Please indicate clearly which points must be addressed to make the article scientifically sound. ( *50 words min.*)

 Background:

 The researchers have given clear information in the background of the study; however need to give more information regarding the list of multimorbidity with heart failure/ coronary heart disease. What is the reason for selecting Depression and anxiety as one of the most common multimorbidities with heart failure or coronary heart disease?. Also, the researchers have mentioned in the title as home-based cardiac rehabilitation. Need more information about home-based CR is this protocol implemented after discharge or before discharge from the hospital? Justify the reason for adding home-based CR. Please write research questions

 Methods:

 Please explain in detail the setting for each work package and also the role of interdisciplinary team members in the home-based CR?.  

 Population: Please clearly specify the population. In phase 3, it is mentioned that a patient with heart failure or coronary heart disease. Clearly specify in the inclusion criteria about the line of treatment, eg, Medical management or surgical management (the treatment protocol will differ for both lines of treatment). Moreover, the treatment decision will differ according to the primary physician, from admission until discharge. Explain the protocol implementation in various centres and countries. Specify the inclusion criteria regarding the cognitive status of the participants- Explain

 Protocol- Explain the validity of the protocol. Who has approved the protocol? Based on what guidelines this protocol developed

 Work package 1: Who will do the screening for depression and anxiety? Explain the process of recruitment, whether the participants will be recruited from the community or hospital.  

 Outcome: Please state the time points the outcome assessment will be used for analysis

 Sample: Define the control group and the difference between treatment groups.

Is the study design appropriate for the research question?

Partly

Is the rationale for, and objectives of, the study clearly described?

Yes

Are sufficient details of the methods provided to allow replication by others?

Partly

Are the datasets clearly presented in a useable and accessible format?

Not applicable

Reviewer Expertise:

I have expertise in the area of heart failure and Cardiac rehabilitation

I confirm that I have read this submission and believe that I have an appropriate level of expertise to confirm that it is of an acceptable scientific standard, however I have significant reservations, as outlined above.

# Reviewer report: Reviewer response for version 1

**DOI:** 10.3310/nihropenres.15169.r35547

**Published:** 2025-06-03

**Authors:** Maria Teresa La Rovere

**Affiliations:** 1 Istituti Clinici Scientifici Maugeri IRCCS, Montescano, Italy

Thank you for giving me the opportunity to revise this study protocol, which outlines a research programme on the implementation of cardiac rehabilitation in low- and middle-income countries (LMICs).

 The proposed study comprises four well-considered work packages: 1) Stakeholder engagement events to co-develop a suitable home-based rehabilitation programme and training materials for providers; 2) An acceptability and feasibility pilot study; 3) A trial involving 3,000 patients with heart disease and comorbid anxiety and depression to evaluate improvement in outcomes over 12 months;  4) Development of a sustainable model for research and rehabilitation delivery.

 The study aims to address the significant disparity in cardiac rehabilitation provision between high-income countries and LMICs.  It seeks to transfer the proven benefits of cardiac rehabilitation to LMICs, which are expected to experience a substantial increase in cardiovascular disease in the coming years. A key strength of the study is its focus on populations with the comorbid conditions of heart disease — anxiety and depression — which are highly prevalent in LMICs. The proposed cardiac rehabilitation approach is clinically

 This study is important, timely and relevant.

 My only observation is that the study does not address gender issues in relation to the development of the rehabilitation programme, the involvement of healthcare staff or the sample size of the effectiveness trial.

**Is the rationale for, and objectives of, the study clearly described?**

 Yes

 The authors provide a precise and comprehensive description of the background to the study and explain how new technologies and the momentum of the pandemic make it possible to implement sustainable rehabilitation programmes, even in LMICs. The study's overall objective and its specific research objectives are clearly stated.

**Is the study design appropriate for the research question?**

 Yes

 A key aspect of the study design is engaging a wide range of stakeholders, including healthcare professionals, providers, patients, and their families, to co-develop culturally sensitive programmes that consider the local context. This co-development process provides the basis for answering the research question regarding the feasibility and acceptability of scaling up the programme to conduct an effectiveness trial. The co-developed home-based rehabilitation program incorporates interventions that have previously been tested in people in the UK. The assessment of feasibility/acceptability within the randomised pilot study includes economic evaluations.

 Strength in the effectiveness trial lies in the adoption of the Win Ratio approach for the analysis of the composite primary outcome.

Is the study design appropriate for the research question?

Yes

Is the rationale for, and objectives of, the study clearly described?

Yes

Are sufficient details of the methods provided to allow replication by others?

Yes

Are the datasets clearly presented in a useable and accessible format?

Not applicable

Reviewer Expertise:

Cardiac rehabilitation

I confirm that I have read this submission and believe that I have an appropriate level of expertise to confirm that it is of an acceptable scientific standard.
